# Supplementary material for: Case–control diagnostic accuracy study of a non-sputum CD38-based TAM-TB test from a single milliliter of blood
Source: Sci Rep. 2021 Jun 23;11:13190. doi: 10.1038/s41598-021-92596-z (PMC8222251; doi:10.1038/s41598-021-92596-z)
Supplement: Supplementary file 1 — Supplementary Information. [file 41598_2021_92596_MOESM1_ESM.docx]

**Supplementary material.** A) The area under the Receiving Operating Characteristics curve (AUC) was extracted to determine that compared to a Median Fluorescence Intensity ratio approach used previously for CD27 [10], the Q2/Q3 ratio and a minimum of five cytokine-producing CD4 T cells were required to optimally diagnose TB in this cohort of presumptive TB patients. B) Risk factors for GeneXpert^®^ positivity and GeneXpert^®^ negativity: Body mass index (BMI); Human immunodeficiency virus (HIV); Unadjusted Odds ratios (ORs); Adjusted Odds ratio (aORs); Logistic regression models (univariate and multivariable logistic regression) was performed to determine association between binary outcome (GeneXpert^®^ positive and GeneXpert^®^ negative) and age in years, sex, body mass index, smoking and HIV status.

A


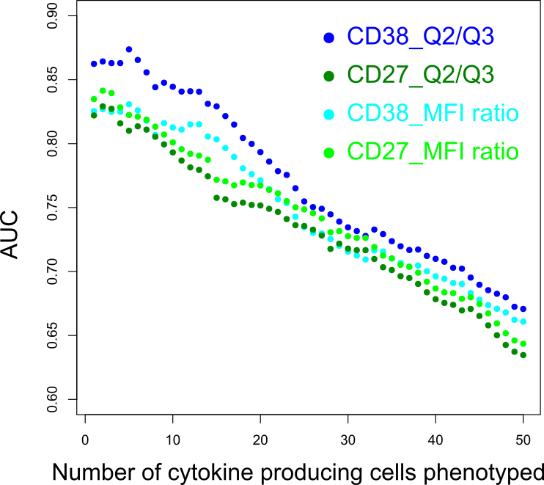


B

| **Characteristics** | **GeneXpert^®^ status** | | **Unadjusted** |  | **Adjusted** |  |
| --- | --- | --- | --- | --- | --- | --- |
|  | **positive, n(%)** | **negative, n (%)** | **OR (95%CI)** | **p-value** | **aOR (95%CI)** | **p value** |
| **Age group (years)** |  |  |  |  |  |  |
| 18-24 | 105 (21.9) | 8 (7.4) | Ref |  | Ref |  |
| 25-44 | 174 (36.3) | 26 (24.1) | 0.94(0.86-1.03) | 0.18 | 0.98 (0.90-1.08) | 0.77 |
| 35-44 | 129 (26.9) | 41 (37.9) | 0.84(0.77-0.92) | 0.001 | 0.89(0.81-0.98) | 0.02 |
| >45 | 71 (14.8) | 33 (30.6) | 0.78(0.71-0.86) | <0.001 | 0.83(0.75-0.92) | 0.001 |
| **Sex** |  |  |  |  |  |  |
| Female | 133 (27.7) | 35 (32.4) | Ref |  | Ref |  |
| Male | 346 (72.3) | 73 (67.6) | 1.04 (0.96-1.11) | 0.33 | 1.05 (0.98-1.13) | 0.16 |
| **BMI category (kg/m^2^)** |  |  |  |  |  |  |
| BMI≥18.5 | 220 (45.9) | 87 (80.1) | Ref |  | Ref |  |
| BMI<18.5 | 259 (54.1) | 21 (19.9) | 1.23(1.16-1.31) | <0.001 | 1.14(1.08-1.21) | <0.001 |
| **Smoking status** |  |  |  |  |  |  |
| no | 368(76.8) | 73(67.6) | Ref |  | Ref |  |
| yes | 111 (23.2) | 35 (32.4) | 0.93(0.86-0.99) | 0.045 | 0.94(0.87-1.02) | 0.12 |
| **HIV status** |  |  |  |  |  |  |
| Negative | 401(83.7) | 82(75.9) | Ref |  | Ref |  |
| Positive | 78 (16.3) | 26 (24.1) | 0.92(0.85-1.00) | 0.055 | 0.95(0.88-1.03) | 0.24 |
